# Supplementary material for: Insights into the cotranscriptional and translational control mechanisms of the Escherichia coli tbpA thiamin pyrophosphate riboswitch
Source: Commun Biol. 2024 Oct 17;7:1345. doi: 10.1038/s42003-024-07008-5 (PMC11487190; doi:10.1038/s42003-024-07008-5)
Supplement: Supplementary file 2 — Supplementary Material [file 42003_2024_7008_MOESM2_ESM.docx]

Insights into the Cotranscriptional and Translational Control Mechanisms of the *Escherichia coli tbpA* thiamin pyrophosphate riboswitch.

**Jonathan P. Grondin^1@^, Mélanie Geffroy^1,2#^, Maxime Simoneau-Roy^1,2†^, Adrien Chauvier^1&^, Pierre Turcotte^1‡^, Patrick St-Pierre^1^, Audrey Dubé^1¥^, Julie Moreau^1^, Eric Massé^2^, Juan Carlos Penedo^3,4^ and Daniel A. Lafontaine^*1^**

^1^Department of Biology, Faculty of Science, Université de Sherbrooke, Sherbrooke, Quebec, Canada.

^2^Department of Biochemistry and Functional Genomics, Université de Sherbrooke, Sherbrooke, Quebec, Canada.

^3^Centre of Biophotonics, Laboratory for Biophysics and Biomolecular Dynamics, SUPA School of Physics and Astronomy, University of St. Andrews, St Andrews, UK.

^4^Centre of Biophotonics, Laboratory for Biophysics and Biomolecular Dynamics, Biomedical Sciences Research Complex, School of Biology, University of St. Andrews, St. Andrews, UK.

**^@^**Present address: Canadian Food Inspection Agency, Ottawa, Ontario, Canada.

^#^Present address: Delpharm Boucherville, Boucherville, Quebec, Canada.

**^†^**Present address: Cégep de Saint-Hyacinthe, Saint-Hyacinthe, Quebec, Canada.

^&^Present address: Single Molecule Analysis Group, Department of Chemistry, University of Michigan, Ann Arbor, MI 48109.

**^¥^**Present address: Unité de recherche clinique et épidémiologique, CIUSSS de l'Estrie, Sherbrooke, Quebec, Canada.

**^π^**Present address: Département de médecine de famille et de médecine d'urgence, Université de Sherbrooke, Sherbrooke, Quebec, Canada.

^*^To whom correspondence should be addressed: daniel.lafontaine@usherbrooke.ca

**SUPPLEMENTARY INFORMATION**

**
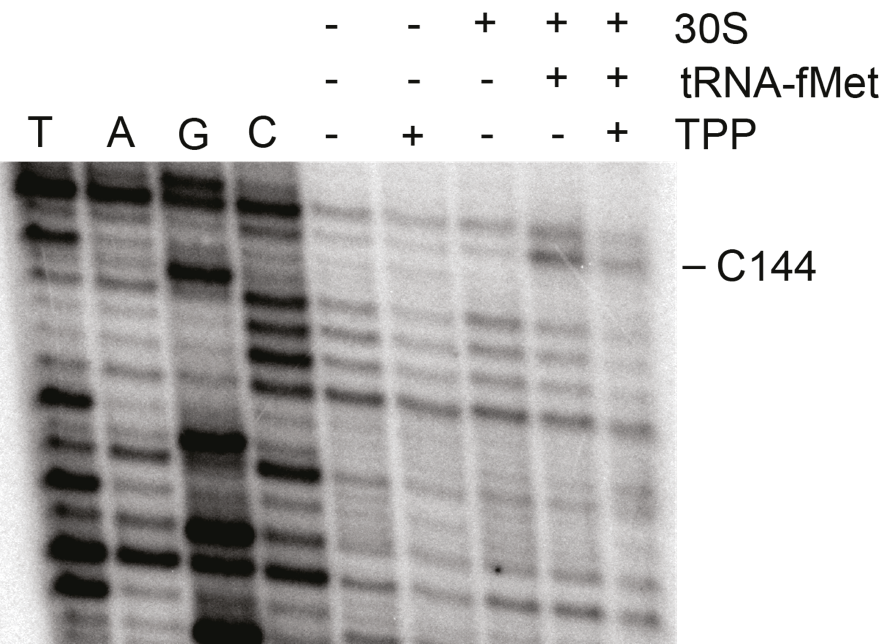
**

**Supplementary Figure 1.** Toeprint assays of the *Escherichia coli tbpA* riboswitch.

Toeprint experiments were performed in the presence of the 30S ribosomal subunit, tRNA-fMet and TPP. The mapping of the toeprint was performed by adding the corresponding ddNTP in the reverse transcription reaction.

**
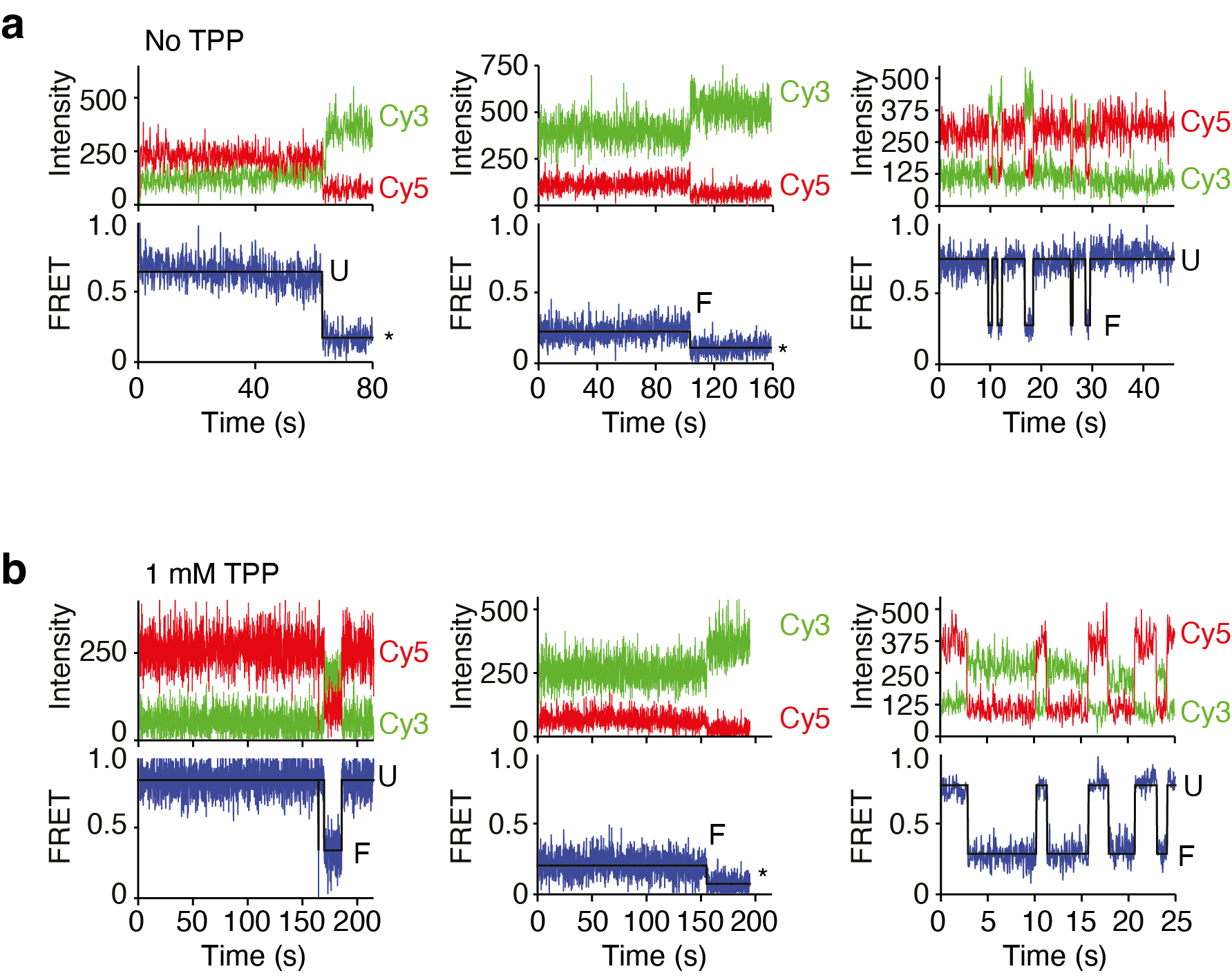
**

**Supplementary Figure 2.** smFRET analysis of semi-synthetic *tbpA* transcripts.

**a,b** Representative smFRET trajectories showing the anti-correlated donor (Cy3, green) and acceptor (Cy5, red) emission intensities, together with the resulting FRET traces. Traces were obtained in the absence (**a**) or presence (**b**) of 1 mM TPP. Black lines represent the hidden Markov modelling of FRET trajectories. The unfolded (U) and folded (F) conformations are identified to the right. Photobleaching events are indicated by asterisks.

**
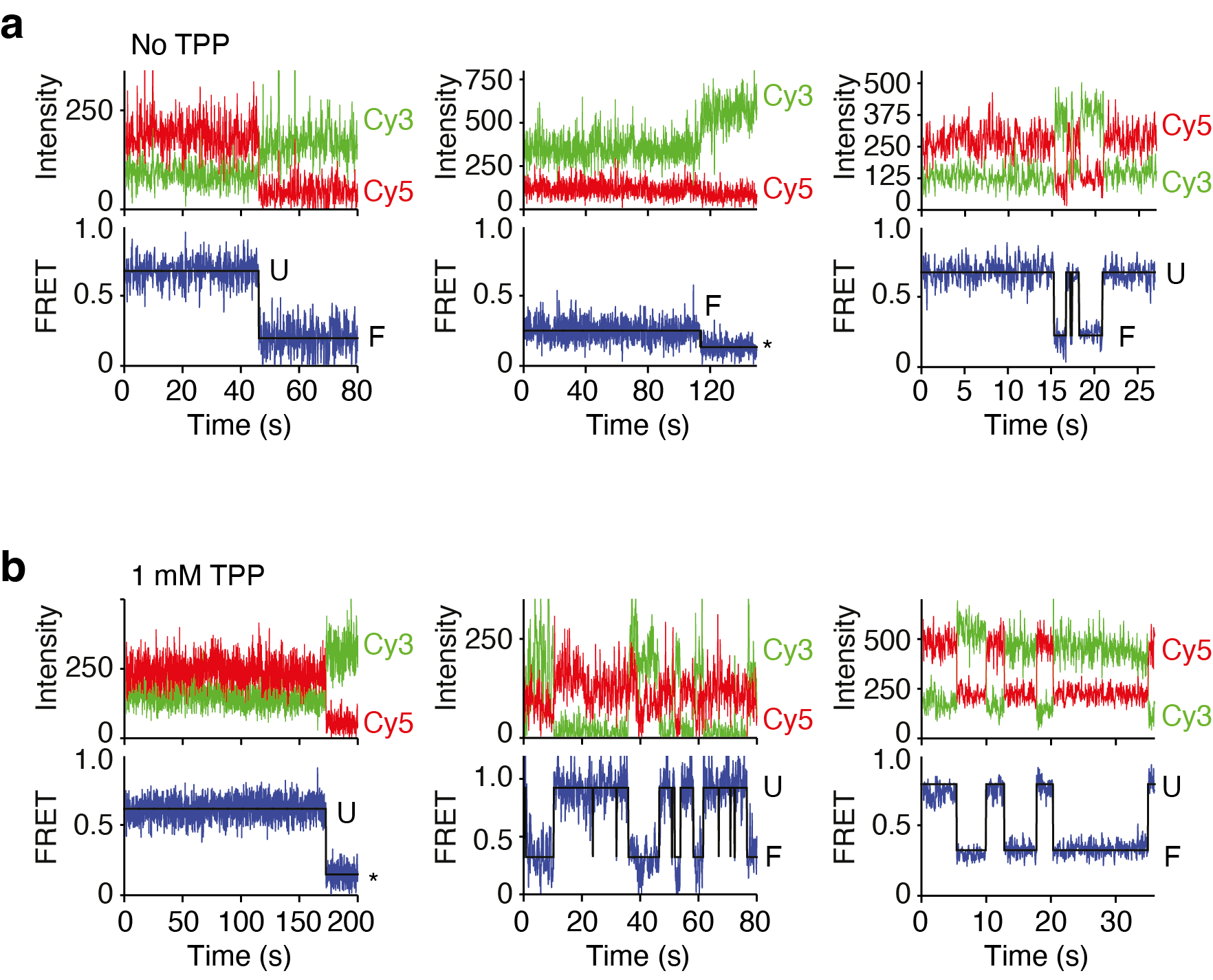
**

**Supplementary Figure 3.** smFRET analysis of nascent *tbpA* transcripts.

**a,b** Representative smFRET trajectories showing the anti-correlated donor (Cy3, green) and acceptor (Cy5, red) emission intensities, together with the resulting FRET traces. Traces were obtained in the absence (**a**) or presence (**b**) of 1 mM TPP. Black lines represent the hidden Markov modelling of FRET trajectories. The unfolded (U) and folded (F) conformations are identified to the right. Photobleaching events are indicated by asterisks.

**
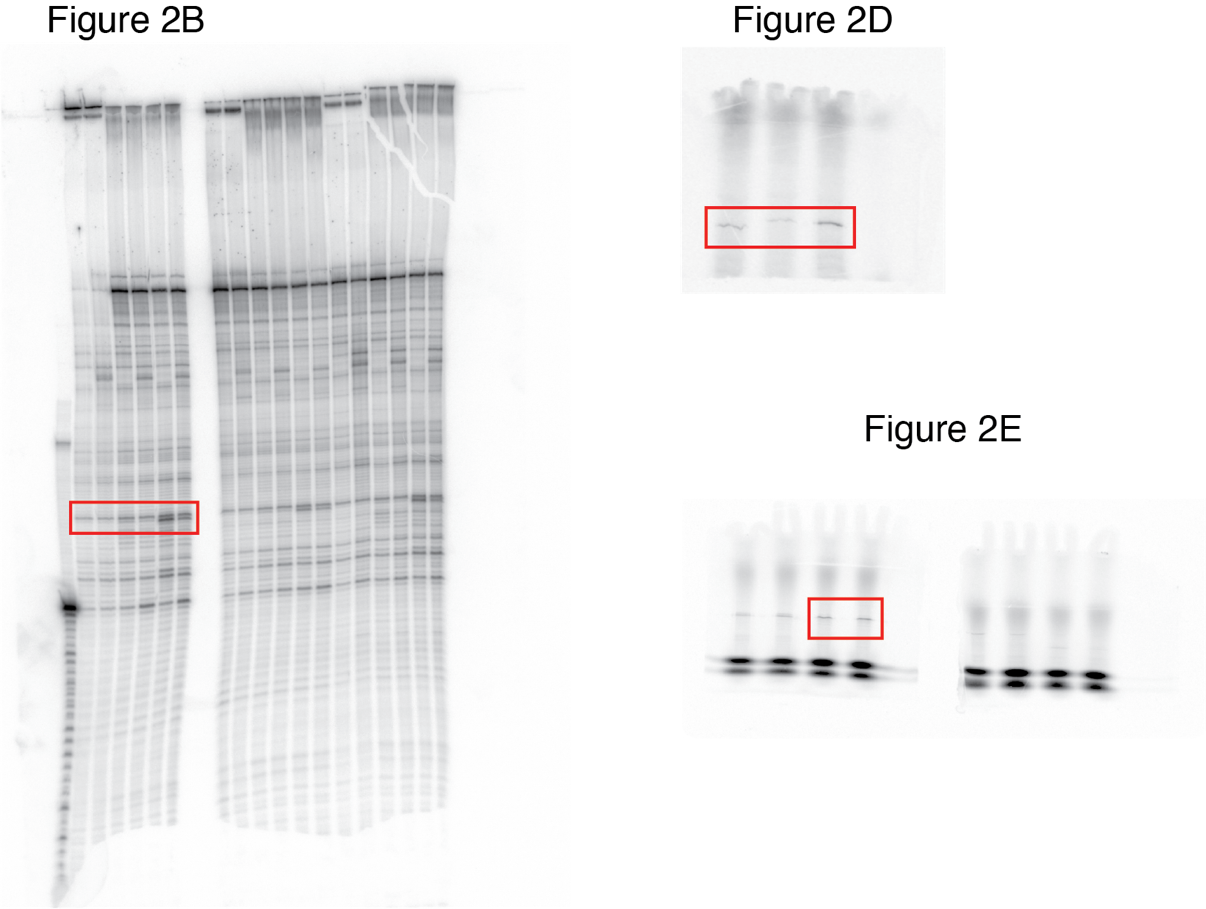
**

**Supplementary Figure 4.** Uncropped figures (Fig. 2B, 2D and 2E). Boxed areas correspond to images presented in the main text.

**
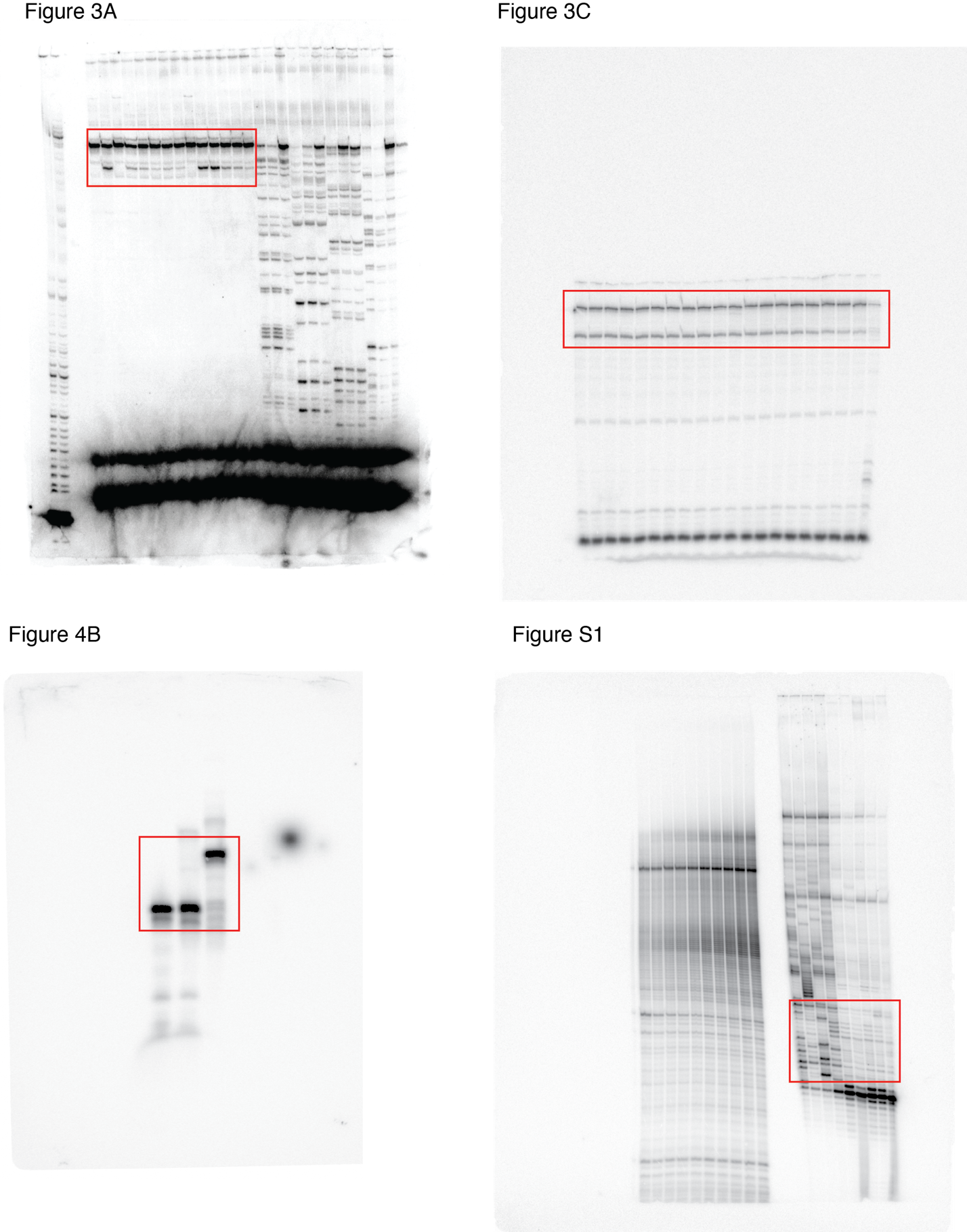
Supplementary Figure 5.** Uncropped figures (Fig. 3A, 3C, 4B and Supplementary Fig. 1). Boxed areas correspond to images presented in the main text and supplementary figures.

**Supplementary Table 1. Summary of strains or plasmids used in this study.**

| **Strains** | **Relevant marker** | **References** |
| --- | --- | --- |
| TrD  TrX  TrD WT  TrD G25C  TrD SD  TrD GAG  TrX WT  TrX G35C  TrX SD  TrX GAG | PM1205 *lacl’*::P_thiB_-ThiB-LacZ  *lacl’*::P_thiB_-*thiB*-LacZ  *lacl’*::P_BAD_-ThiB-LacZ  *lacl’*::P_BAD_-ThiB-G35C-LacZ  *lacl’*::P_BAD_-ThiB-G121C-LacZ  *lacl’*::P_BAD_-ThiB-U130A-LacZ  *lacl’*::P_BAD_-*thiB*-LacZ  *lacl’*::P_BAD_-*thiB*-G35C-LacZ  *lacl’*::P_BAD_-*thiB*-G121C-LacZ  *lacl’*::P_BAD_-*thiB*-U130A-LacZ | This study  This study  This study  This study  This study  This study  This study  This study  This study  This study |

**Supplementary Table 2. Summary of *lacZ* fusions used in this study.**

| **Strains** | **Constructs** | **Oligonucleotides** |
| --- | --- | --- |
| TPP1 | TbpA_12cd_ | MSR8-MSR6 (genomic DNA) |
| TPP2 | P_NAT_-*tbpA* | MSR8-MSR9 (genomic DNA) |
| TPP3 | P_BAD_-thiB-LacZ | MSR5-MSR9 (genomic DNA) |
| TPP4 | P_BAD_-thiB-G25C-LacZ | MSR5-MSR91 + MSR90-MSR6 |
| TPP5 | P_BAD_-thiB-G121C-LacZ | MSR5-MSR59 + MSR60-MSR6 |
| TPP6 | P_BAD_-thiB-U130A-LacZ | MSR5-MSR61 + MSR62-MSR6 |
| TPP7 | P_BAD_-*thiB*-LacZ | MSR5-MSR7 (genomic DNA) |
| TPP8 | P_BAD_-*thiB*-G35C-LacZ | MSR5-MSR91 + MSR90-MSR7 |
| TPP9 | P_BAD_-*thiB*-G121C-LacZ | MSR5-MSR59 + MSR60-MSR7 |
| TPP10 | P_BAD_-*thiB*-U130A-LacZ | MSR5-MSR61 + MSR62-MSR7 |
|  |  |  |

**Supplementary Table 3. PCR constructs used for *in vitro* RNA synthesis.**

| **Constructions** |  | **Oligonucleotides** |
| --- | --- | --- |
| ***In vitro* transcription assays** | |  |
|  | |  |
| *pLacUV5-tbpA-88-extBio* | | 275AL-2439AC (genomic DNA) |
|  | |  |
| ***In vitro* transcription-translation assays**  *pLacUV5-lacZ*  *pLacUV5-tbpA_306cd_* | | PCR1: 988MG-986MG (genomic DNA)  PCR2: 989MG-986MG (PCR1)  275AL-2666AC (genomic DNA) |
| **Transcription elongation complex**  *pLacUV5-tbpA-EC-136*  *pLacUV5-tbpA-EC-180*  *pLacUV5-tbpA-EC-248*  **T7 RNAP transcription**  *pT7-tbpA-EC-180*  *3’ tbpA-89* | | 275AL-1575JFN (genomic DNA)  275AL-2759JG (genomic DNA)  275AL-3836JG (genomic DNA)  3360EH-2759JG (genomic DNA)  1987PSTP-1988PTSP (genomic DNA) |

**Supplementary Table 4. Oligonucleotides used in this study.**

| **Oligonucleotides** | **Sequences 5'-3'** |
| --- | --- |

| **LacZ fusions** |  |
| --- | --- |
| MSR 5 | ACCTGACGCTTTTTATCGCAACTCTCTACTGTTTCTCCATGTTCT  CAACGGGGTGCCAC |
| MSR 6 | TAACGCCAGGGTTTTCCCAGTCACGACGTTGTAAAACGACGCAC  AGCAACAGCAGGGG |
| MSR 7 | TAACGCCAGGGTTTTCCCAGTCACGACGTTGTAAAACGACCATA  GCTGTTTCCTGTGTGAGCACAGCAACAGCAGGGG |
| MSR 8 | ACCTCGACGCTTTTTATCGCAAC |
| MSR 9 | TAACGCCAGGGTTTTCCCAGTCACGACGTTGTAAAACGACCATA  GCTGTTTCCTGTGTGAGTGGCACCCCGTTGAGAAC |
| MSR59 | CATTTTTTTAACACTTTGCACGTCAAAAAAGAGTGGC |
| MSR60 | GCCACTCTTTTTTGACGTGCAAAGTGTTAAAAAAATG |
| MSR61 | CAGACATTTTTTTAACTCTTTGCACCTCAAAAAAG |
| MSR62 | CTTTTTTGAGGTGCAAAGAGTTAAAAAAATGTCTG |
| MSR90 | GCGTACGCGTGCGCTCAGAAAATACCCGTCG |
| MSR91 | CGACGGGTATTTTCTGAGCGCACGCGTACGC |
|  |  |
| **Toeprint** |  |
| 275 AL | GGGCACCCCAGGCTTTACACTTTATGCTTCCGGCTCGTATAATG  TGTGGCTGACCGCCAGGAGTGGAT |
| 2759JG | CGGGTTTAGCGAAAACGGGC |
|  |  |
| **T7 RNAP constructs** |  |
| 2558JG | 5’Cy3-GUUCUCAACGGGG-iAzideN-GCCACGCGUACGCGUGCGCUGAGA |
| 3360EH | GTAATACGACTCACTATAGCGTTCTCAACGGGGTGCCAC |
| 1987PTSP | GGTAATACGACTCACTATAGGGTATTTCTGATGAGGCCTTCGGGCCGA  AACGGTGAAAGCCGTAAAATACCC |
| 1988PTSP | GGCCGGGGGGCCGGGGAGCCTCAAATCCCTTCGCCGGCGTTATCCGGA  TCAGGTTCGACGGGTATTTTACGGCTTTCACCGTTTCGGCC |
|  |  |
| **In vitro translation** |  |
| 986MG | AAACCCCTCCGTTTAGAGAGGGGTTATGCTAGTTACATCTGAACTTCAG  CCTCCAGTAC |
| 988MG | GGGCTTAAGTATAAGGAGGAAAAAATATGGTCGTTTTACAACGTCGTG  ACTG |
| 989MG | GGGCACCCCAGGCTTTACACTTTATGCTTCCGGCTCGTATAATGTGTGGG  GGCTTAAGTATAAGGAGGAAAAAATATG |
| 2666AC | AAACCCCTCCGTTTAGAGAGGGGTTATGCTAGTTCGTGAACTCCAACGTG  GTTGC |
|  |  |
| **Elongation complexes** |  |
| 1575JFN | 5’biotin-AGACATTTTTTTAACACTTTGCACCTC |
| 2439AC | agaccacgttgaaagattgggtttgGAGCCTCAAATCCCTTCGCCG |
| 2759JG | 5’biotin-CGGGTTTAGCGAAAACGGGC |
| 3836JG | 5’biotin-GGCTTTTTTAACCACCGGAC |
|  |  |
| **RNase H** |  |
| 923AC | TTTGCACCTC |
| 2477AC | GACGGGTATT |
|  |  |
| **DNA Anchors** |  |
| 2344AC | 5’biotin-AGACCACGTTGAAAGATTGGGTTTG |
| 2559JG | 5’biotin-GGCCGGGGGGCCGGGG |
|  |  |
