# Supplementary material for: Insights into the cotranscriptional and translational control mechanisms of the Escherichia coli tbpA thiamin pyrophosphate riboswitch
Source: Commun Biol. 2024 Oct 17;7:1345. doi: 10.1038/s42003-024-07008-5 (PMC11487190; doi:10.1038/s42003-024-07008-5)
Supplement: Supplementary file 3 — Description of Additional Supplementary Files [file 42003_2024_7008_MOESM3_ESM.pdf]

Description of Additional Supplementary Files

File name: Supplementary Data

Description: The source data behind the graphs in the paper.
